# Creativity and Cognition in Extreme Environments: The Space Arts as a Case Study

**DOI:** 10.3389/fpsyg.2020.575291

**Published:** 2020-09-29

**Authors:** Kathryn Hays, Cris Kubli, Roger Malina

**Affiliations:** ^1^Department of Information Sciences, University of North Texas, Denton, TX, United States; ^2^Department of Brain and Behavioral Sciences, University of Texas at Dallas, Richardson, TX, United States; ^3^Department of Arts, Technology, and Emerging Communication, University of Texas at Dallas, Richardson, TX, United States

**Keywords:** space art, extreme environments, creativity, 4E cognition, astronomical art, astronautical art, social innovation

## Abstract

Humans, like all organisms, have evolved to survive in specific environments, while some elect or are forced to live and work in extreme environments. Understanding cognition as it relates to environmental conditions, we use 4E cognition as a framework to explore creativity in extreme environments. Our paper examines space arts as a case study through the history, present practices, and future possible arts in the context of humans beyond the Kármán boundary of the Earth’s atmosphere. We develop a proposed taxonomy of space arts, based on prior taxonomies, and provide specific exemplars of space art developed by artists in space or for use by astronauts in space. Using examples of space art since the birth of the space age, we discuss (1) how human survival in extreme environments requires investment in the space arts, driven by consideration of various biopsychosocial factors and (2) how new scientific and engineering discoveries; such as the detection of air current patterns with paper airplanes in zero gravity, could be consequences or examples of creative thinking driven by artists in the various types of space art. We conclude by discussing possible benefits of space art, future research applications, and advocate that all space actors, government or private, involve artists in all projects beyond the Kármán Boundary of the Earth’s atmosphere.

## Introduction

Beyond aesthetic or communicative functions, art serves as a tool to provoke new perspectives on exploration and introspection, both on an individual or societal scale. Art is work intended to stimulate emotions, through either their perception or comprehension (Malina, 1970). The process of making or interacting with art utilizes embodied cognition to engage with the environment and provides insight into other cognitive processes (Ticini etal., 2015; Silver, 2013). Studying human performance in extreme environments can enable comprehension of and planning for activity in future extreme environments, such as off planet colonies and deep space missions with no contact. We seek to understand how extreme environments may enable the generation of novel ideas that may not occur in another context through examination of the history of space art, in addition to understanding how this extreme environment impacts cognition and creativity. To augment the limited research on creativity in space, we argue for the utilization of research from other extreme environments with similar contextual factors. We give examples and discuss creative thinking driven by artists that generates new scientific and engineering discoveries. Contemporary transdisciplinary approaches (Mejía et al., 2018) are paramount in this chapter, as the understanding of cognition as it relates to environmental factors bridges the relationship between creative ideation and extreme environments like space. Moreover, this approach can inform current and future practices for space activities, as well as applications of insight generated from space art practices.

### Extreme Environments

Humans live, adapt, and understand their experience through environmental context. While humans have evolved to thrive in specific Earth environments, some elect to or are forced to live in extreme environments. Extreme environments are contexts with demanding physiological and psychological conditions, beyond an optimal range, that affect cognition, behavior, physiology and genetics (Paulus et al., 2009; Ilardo and Nielsen, 2018). Examples of these environments include outer space, deep ocean, sustained extreme temperatures, and isolation. Changes in context prompt adaptive changes in cognition and behavior; extreme environments exacerbate this by disturbing physiological and psychological states, prompting complex cognitive and affective responses (Paulus et al., 2009).

Changes that occur when adapting to space environments is a decades old topic of research, funded and documented by international space agencies and non-affiliated researchers alike. This research spans many topics including changes in physiological states, like blood pressure and circulation, sensory deficits, or vestibular sense (e.g., Buckey et al., 1996; Clément and Reschke, 2008; Hallgren et al., 2016); cognitive changes, like sensorimotor deficits, attention and cognitive functioning, or effects from disturbances in circadian rhythm (e.g., Palinkas, 1991; Bock, 1998; Liu et al., 2015); and emotional changes, like depression, factors of interpersonal conflict, or approaches to improving mental health (e.g., Palinkas, 2003; Ritsher, 2003; Salamon et al., 2017).

It is important to acknowledge the many factors that make space uniquely extreme, such as the risk of radiation, variable gravity forces, the lack of a diurnal cycle, and distance from Earth with limited recourse in case of an emergency, that exacerbate the other features of the extreme environment. We insist that living in space is not ‘like Antarctica but further’ because the other extreme environments on Earth do not present these particular compounding challenges and it is yet to be observed how humans adapt to or handle such hazards long term.

While transitioning to a zero-gravity environment is a challenging process, both physically and mentally, transitioning between environments causes a reevaluation of events, actions, and demands, which has a higher potential to evoke new and original ideas (Runco and Charles, 1993). It provides an opportunity to reevaluate features in the environment and question previous assumptions, which is strongly related to the creative process (Amabile and Gryskiewicz, 1987). Being in the zero gravity environment of space compels individuals to engage in these procedures and results in creative ideation and the creation of artworks in or about their experiences in space.

## Space Art

The first space art is commonly identified as the intentional and realistic depiction of space flight by Emile-Antoine Bayard and Alphonse-Marie de Neuville for Jules Verne’s novel, *From the Earth to the Moon* in 1865. However, this neglects the centuries of art that clearly depicts astronomical phenomena or even art that may have at its time been intended to be viewed from the heavens (e.g., Nazca lines in Peru, lunar petroglyphs at Ngaut Ngaut). These artists are clearly inspired by astronomical discoveries and phenomena combined with their own creativity.

The International Association of Astronomical Artists (IAAA) gathers current space and astronomical art practitioners. In this paper, we chose to differentiate between the astronomical arts that continue as contemporary arts and the space arts enabled by the first successful launch of objects and/or humans into space. We use the Kármán line (100 km above mean sea level) as a definition of where outer space “begins,” as endorsed by the International Astronautical Federation; we note that other definitions exist.

The IAAA definition states: “…the genre of Space Art itself is still in its infancy, having begun only when humanity gained the ability to look off our world and artistically depicted what we see out there Szathmary (2020).” Malina (1991) defined space art as “contemporary art which relies for its implementation on participation in space activity;” Arthur Woods (2019) chose to use the term “Astronautical Art” which we adopt here. In this definition space arts include artforms created above the Kármán line, but also artforms enabled by space vehicles, e.g., telecommunication satellites. Artforms enabled by “spin off” space technologies (e.g., Teflon) are not space art in this definition.

We argue therefore that space art can incorporate elements of both astronomical art and astronautical art. Astronomical art focuses on conceptualizing and visualizing outer space phenomena, whereas astronautical art relies on outer space environments or technology for its actualization (Woods, 2019). The IAAA maintains a similar distinction: “Space Art” is inspired and generated from space based knowledge and ideas, like astronomical art, and “Art In Space,” utilizes space conditions and environment as a component or tool, like astronautical art. These categories do not create a dichotomy, rather they have a significant amount of overlap.

Space art is an extension of environmental and land art movements (Malina, 1991); artists appropriate the natural world both as materials for making art and as the source of ideas and concepts. Space art seeks to explore outer space using projects that rely on space technologies, materials, or environment for their realization (Woods, 2019).

### Taxonomy

**FIGURE 1:**
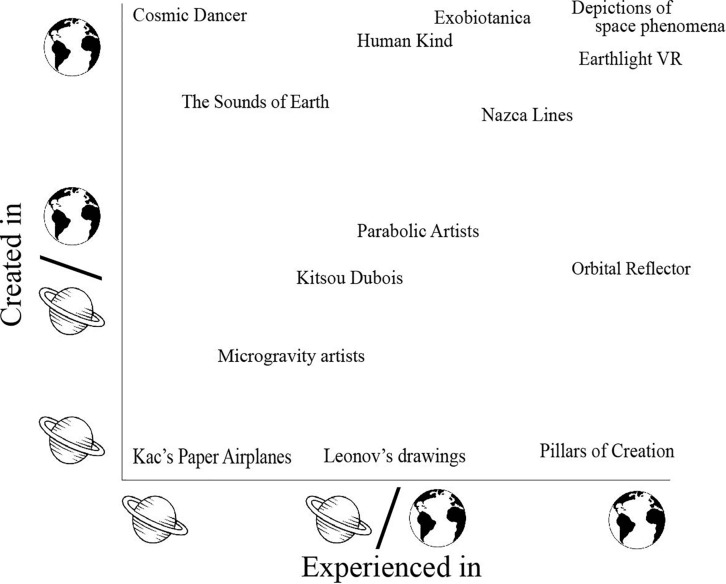
Visualization of space art examples on two dimensions displays the spectrum of contexts for both creation and experience.

To communicate and assess different artforms in the context of space, we present a taxonomy for space art that is based on taxonomies to explain different forms of space art (Malina, 1991; Woods, 2019; Bureaud, 2020). Our taxonomy is highly reliant on two dimensions: where the art is created and where it is experienced (Figure 1). We incorporate a category for “astronomical art” that captures artworks that depict space phenomena (Woods, 2019) and are created and experienced on Earth. Our taxonomy provides exemplars of each category, spanning many artforms and practices (Table 1). It must be noted that due to the limited existing works of space art, some categories have few documented artworks, especially those created in extreme environments.

**TABLE 1:** Space art taxonomy with exemplars.

	**Space art**							
	**Astronomical**	**Astronautical**						
Contextual definition	Art depicting or imagining space phenomena	Created outside EE of Space Experienced in both	Created outside EE of Space Experienced in EE	Created in EE Experienced in EE	Created in both Experienced in both	Created in EE Experienced outside	Created in EE Experienced in both	Art in a simulated ZG env., eg parabolic flights
Exemplar	Nebra sky disc (1600 B.C.E.)	Tesla Roadster “Starman”	Wood’s “Cosmic Dancer”	Kac’s paper airplanes, “Inner Telescope”	“ArtSat” Kriesche	Trevor Paglen’s “Orbital Reflector”	Cupula professional photography of Earth	Kitsou Dubois’s choreography
Exemplar	Instrument: One Antarctic Night	“Fallen Astronaut” on Moon (Apollo 15)	Pierre comte’s Prisma	Water marbling, Osaka Furukawa	“I.S.S. Is somebody singing?” Duet	Chris Hadfield’s “Space Oddity” Music Video	Scott Kelly’s gorilla costume chase	Pietronigro’s Drift Paintings

## Cognition and Creativity

Art making is aided by global and local context features (Brinck, 2007). In this sense, creative undertakings can be presented as complex cognitive processes that are highly interdependent on the environment where the artist is located. We present cognition and creativity using 4E cognition as a framework.

### Situated Cognition

Situated cognition is based on the notion that cognition is tied to external factors like context, action, and language (Smith and Semin, 2004). This theory is based on context-sensitive cognition, which heavily relies on environmental factors (Schwarz, 2006). Interdisciplinary theories like situated cognition are regularly considered in many social science subfields like environmental psychology and ecological anthropology.

The environment acts as a medium for action and interaction (Sosa and Gero, 2003). Human cognition encourages situational adaptation based on evaluation from given affective and environmental cues. Safe and familiar situations elicit top-down processing and reliance on routine behavior, while unfamiliar or problematic situations would evoke systematic, bottom-up processing and detail-oriented attention (Schwarz, 2002). In unfamiliar situations, actions and contexts are represented in more detail (Wegner et al., 1986). Cognitive tuning, and related bodily sensations, also provide cues to the nature of a situation (Friedman and Förster, 2000), meaning that bodily responses and physical sensations can evoke heuristics, processing styles, and situational adaptations. As cognition can adapt to a given context or environment, cognitive operations, like creative ideation, will vary in the same way. By understanding how creativity occurs in a given context, we can better understand how to evoke and facilitate novel ideas that wouldn’t occur in a different environment.

### 4E Cognition

Cognition is related to the environment, therefore the consideration of environmental or task context-needs enables the facilitation of cognitive processes, such as creativity, beyond those needed for survival and safety. This process is expedited through the development of context-sensitive, cognitively informed training. The 4E theory of cognition posits that cognition is embodied, enacted, embedded, and extended (Rowlands, 2010; Newen et al., 2018). This approach provides a framework for the relationship between cognition, creativity, and the environment.

• Embodied cognition is the theory that cognition is extra-neural and originates in physical interactions that rely upon sensorimotor abilities (Smith and Semin, 2004). Physical movements and interactions have an observed impact on cognitive processes (Schwarz, 2006) and styles (Friedman and Förster, 2000).
• Enacted cognition states that cognition functions to serve action (Schwarz, 2006). This is enabled by evaluation and responsiveness to physical environments and social contexts, as well as the context-sensitive activation of knowledge (Avnet and Higgins, 2006).
• Embedded cognition is the notion that cognition is dependent on the relationship with the environment and the social context (Ward and Stapleton, 2012). This theory is closely related, and at times viewed as a subcategory, to embodied cognition (van de Laar and de Regt, 2008).
• Extended cognition is the view that cognition is not only embodied, but extended to the environment (Clark and Chalmers, 1998). This is based on the theory of distributed cognition (Hutchins, 1995, 2000), which views cognition as not solely occurring in the mind but across objects, people, and time (Glăveanu, 2012).

4E cognition is characterized by the dependence and interaction with environment artifacts and tools (Kono, 2010; Glăveanu, 2012). The notion that cognition is inherently related to and can be offloaded onto the environment relates to context-sensitive cognition, in that the use of environmental artifacts facilitate cognitive styles and lessen cognitive load. Again, as cognition is related to and altered by the environment, so are cognitive operations and processes, like creativity.

### Creativity

Creativity, or the production of novel ideas deemed useful and situationally appropriate (Stein, 1974; Amabile, 1983, 1996; Mumford and Gustafson, 1988; Sternberg, 1999), is invaluable to generating new knowledge and scientific insight. Research literature provides many related theories and models to understanding or generating creativity in the context of a domain or field (Feldhusen and Goh, 1995; Sternberg, 1999; Isaksen and Lauer, 2001; Batey and Furnham, 2006).

Creativity is stimulated and evaluated by multiple factors, like cognitive ability, personality factors, knowledge, and environment (Csikszentmihalyi, 1999; Moss, 2002; Batey and Furnham, 2006; Stein, 2014). Research on creativity is based on ideation, regardless of the idea type, reasons and causes, or source of the process (Unsworth, 2001). Both divergent and convergent thinking styles play a part in creative ideation (Runco, 1991; Cropley, 2006). Approaches like pluralism and multivariate theories combine theories, including cognitive, emotional, and environmental, that each have different methods and levels of analysis to provide a more robust understanding of creativity (Kaufman and Sternberg, 2010; Kirsch and Houssemand, 2012; Nelson and Botella, 2017).

Cognitive theories of creativity rely upon psychological research and methodologies (Finke et al., 1992; Smith et al., 1995; Ward et al., 1999) to understand creativity, and reject creativity as a mysterious, unobservable, or fundamentally unusual (Kaufman and Sternberg, 2010). This approach is used to evaluate the nature of cognitive processes related to knowledge, memory, and ideation. It contributes to the existing notion that knowledge impacts the process and outcome of creativity (Mumford and Gustafson, 1988; Sternberg and Lubart, 1995; Cropley, 1999; Feldhusen, 2002; Kaufman and Sternberg, 2010) and through utilization of knowledge, creativity increases its value (Kao, 1997). Theories that overemphasize the role of knowledge can mistakenly suggest a parallel definition of creativity with intelligence. Creativity is not limited to cognitive functioning, but is shaped by cognition, personality traits, environmental conditions, and sociocultural factors (Feldhusen and Goh, 1995).

Based on the notion of situated cognition, it is crucial to evaluate the situated factors that affect creativity (Sosa and Gero, 2003; Gervais et al., 2013). This allows its conceptualization to develop beyond simply a set of cognitive operations (Finke, 1996) and integrates the cognitive process and application of the idea (Nonaka and Zhu, 2012; Wright, 2016). Using situated cognition as a lens accentuates the domain specificity of creativity (Plucker and Beghetto, 2004), while recognizing generalized and task-specific aspects (Lubart and Guignard, 2004). Situational discretion in the use of creative thinking has been tied to psychological traits, like agency and autonomy (Gervais et al., 2013). Many creativity metrics use environmental dimensions, like context and climate, to evaluate the impact on the creative process and output (e.g., Amabile, 1996; Ekvall, 1996, 2000). Distributed creativity acknowledges the sociocultural and embedded nature of creativity and artistic expression, reiterating the value of considering not only the cognitive operations and processes, but also the larger context (Harth, 2004; Glăveanu, 2014).

Examining creativity in a situated manner, informed by a 4E cognition framework, provides a complement to personality and domain driven research. Just as people shape and alter their environments to support their activities and endeavors, the environment provides scaffolding for physical, cognitive, and sociocultural actions (Brinck, 2007). This further solidifies extended and embedded cognition through use of tools and environmental artifacts to augment limited cognitive capacities (Schwarz, 2006).

### Sociocultural Interdependencies

As 4E cognition expands cognitive theories on creativity to include context and other environmental factors, the incorporation of anthropological perspective furthers this expansion to include sociocultural elements. Creativity is a social process (Grossen, 2008), that occurs within the context of relationships and dialogues (Barrett, 1999; Glăveanu, 2012). Evaluating creative processes and practices between and within cultures informs our expectations for creative behavior that is situated in the most extreme environments. The appreciation of aesthetics is multifaceted; it can be relative to an individual’s personal, professional, or cultural background (Crowley, 1958), or dependent on the participation and the shared experience of making art (DeMarrais and Robb, 2013).

Aubert et al. (2019) published a finding of a cave painting in Maros, Indonesia, which is disputed as the oldest finding of this category dated back to at least 43,900 years. While cave paintings may be considered as parietal art or prehistoric art, it is difficult to investigate whether paleolithic societies considered their works as art, or if they even had a concept of “art.” Renowned anthropologist Geertz (1976) describes art as being part of a cultural system, which consists of “inherited conceptions expressed in symbolic forms” used to communicate and develop knowledge about life (Geertz, 1973, p. 89). It is recognizable that such ancient cave illustrations, regardless if they are considered as art, are indeed creative and symbolic practices. While art reflects creativity, creativity does not always produce art.

Anthropology focuses on understanding ourselves and inner perceptions of society, often through comparison. Western art practice regularly institutionalizes art and often treats it as an independent entity, opposed to societies where artistic practices are a component of everyday life. Some governments incorporate some type of ministry related to art and culture, in addition to museums dedicated to specific types of art. In these societies, making art is a plausible option as a career path, to the point where it can be monetized, yet it is still susceptible to critics and tied to the sociocultural context of that time. Meanwhile, less industrialized societies have definitions without fine distinctions and artists may not be able to subsist by their creative work. Rather, their work is guided by responsibilities within their community.

Even though art in its many forms is tied to religion directly or indirectly, it must be noted that less industrialized societies, – where religion is a strong component in everyday life – might value the artist’s creative and individual performance more than the religious undertones themselves (Osborne and Tanner, 2008).

There are several divisions when it comes to understanding art, but irrespective of the economic value or specific practices across societies, creativity is universally present. Creativity can improve well-being, communication skills, and interpersonal collaborations – even problem solving in unusual or dangerous situations (i.e., King, 1997; Bourgeois-Bougrine and Lubart, 2019).

## Comparison With Other Extreme Environments

Due to the novelty of space travel itself and the cost of researching in space, the amount of extant research on individuals and groups in space is limited. Therefore, the use of space analogs and other extreme environments can provide predictive insights (Bishop, 2013).

Cultural and biological alterations increase human adaptability to include extreme environmental conditions. To date, there are well-established cities in areas with extreme temperatures such as polar regions or deserts (ranging from −40 to 40°C, respectively), and with high altitude (over 3,000 meters above sea level) despite the possible health concerns for those living in these surroundings. For example, in the Antarctic there are a diverse set of temporary and permanent camps. A few people have been born in those camps and researchers detect a shift in phonetics, suggesting that the inhabitants are developing their own accent (Harrington et al., 2019). This linguistic development may occur in the analog of space. Novel communication methods and shared terminology is necessary for unknown phenomena specific to the context and to aid collaborative efforts.

Johnson et al. (2003) emphasized that participants on polar expeditions are physically isolated from the outside world, with darkness and weather conditions exerting severe restrictions on travel and separation from families and friends. In addition, there is little separation between work and leisure during such expeditions because living and working spaces are close to one another, and interactions are with the same individuals during both activities. This constant interaction is reported to often create increased social conflict between workers and supervisors, coworkers, cliques, and people with conflicting personalities (Johnson et al., 2003). A significant subset of individuals who spend extended periods of time in polar settings experience depression, insomnia, irritability, and hostility (Gunderson, 1968).

After long term exposure to desert heat, decreased psychomotor speed, attentional and executive functions, and overall cognitive performance were observed (Maruff et al., 2006). Deep divers confined for long periods in a hyperbaric chamber reported anxiety co-occurring with low self-control and emotional instability (Abraini et al., 1998). Individuals in nuclear submarines experienced interpersonal friction, monotony, and lowered morale and motivation (Flinn et al., 1961). While there are overlaps in qualities of environments that enable cross application, it is crucial to acknowledge the variance between each extreme environment.

Given the limited social science research in zero gravity and outer space, specifically on creativity in space, we argue for the use of research from other extreme environments with similar contextual factors. Consideration of other habitable extreme environments where humans have had a long presence could potentially inform on the experience of space flight and the necessary considerations for future space activities.

## Applied Social Research for Space

Future space activities can be furthered by considering cognitive and social approaches, since these activities are guided by and designed for humans. The implementation of cultural, social, and psychological perspectives is invaluable for studies of humans in extreme environments, particularly space as it is a setting completely foreign to any earthling since their evolutionary path is unique to our planet. Wherever humans go, they take their biological and social components with them.

It is important to note that humans, just like other organisms, have a high capacity for adaptability that is aided by current technological advancements, making life possible in even the most unyielding surroundings. Technology as crucial as space shuttle life support systems is developed on Earth, which guides its use in the harsh conditions of space and the disorientation of zero gravity (Trusch, 1982). This type of issue can be improved through cognitively informed testing using tools, for example in a simulated weightless environment (Aoki et al., 2005).

Sociocultural research in zero gravity is a sparse field, as this research is time and effort intensive. It is often underfunded and at times underappreciated by other experts concentrating in space efforts. Scholars have found that, while considering sociocultural and interpersonal aspects of long-term space flight, there have been variations in interpersonal conflict between genders and cultural groups (Palinkas, 1991; Kanas and Caldwell, 2000). Factors that had an observed impact on interpersonal relationships were group tension, cohesion, leadership styles, and group diversity (Palinkas, 2003). Issues like the interaction between crewmates and the design of space environments necessitate a socially informed, human centered approach. Traditionally, the field of human factors, or ergonomics, considers cognitive and biological factors for the design of complex technology. However, a more holistic view would be advantageous. Integrating the biological, psychological and sociocultural elements in equal parts would develop an improved design that is inclusive and accessible. Such insights gained from this type of research may aid in the design of space stations and enhance communication efforts in heterogeneous extraterrestrial settings.

## Discussion

Both art and creativity are complex topics that differ between fields, scholars, and creators. Creative thinking is an inherent human process that results in new, innovative ideas and enables an individual or a group to overcome both spontaneous and long-standing hurdles. Despite the plethora of information and practices concerning creativity in general, we emphasize the wrong word nature of creativity as it relates to space activity and its potential in the not-so-distant future.

While human spaceflight expands and becomes more accessible −as seen recently with the DM-2 mission by SpaceX where commercial spaceflight was inaugurated−, it is crucial to understand the complete set of factors that may limit or enhance creative thinking. Transitioning into a novel environment triggers innovative thinking (Runco, 2003), yet the novelty of space leaves many of these transitions uninspected, for example the fluctuation of gravitational forces or the variation of creative ideation during a spaceflight.

We touch on three different perspectives for studying creative thinking and practices in variable gravity: (1) the biological perspective includes physiological, evolutionary, and adaptive factors; (2) the individual perspective, framed by the 4E cognition theory, is highly dependent on the interaction between the person and its environment; (3) the sociocultural perspective contextualizes creativity as a social process, shaped by social biases, collaboration, and communication.

One thing is quite certain, the human factor cannot be stripped from any activity in space. We emphasize the value of creativity to the human experience and the unique characteristics of space as an extreme environment. Space art diverges from traditional art in the sense that it has major implications resulting from its complex nature as discussed earlier, while simultaneously originating from unimaginable distances. The presented taxonomy attempts to clarify this via 8 specific contexts of space art. Fostering artistic activities that are common on Earth should be approached in the same manner in space to drive both creativity, innovation, and novel scientific discoveries.

### Methodological Integration

There are countless stories of creative practices during times of physical and psychological strain, such as the fruitful artistic life of Nazi-occupied Paris (Riding, 2011) or murals depicting heroic first responders (Lomelí, 2018; Jeffery, 2020). Artists and creators may purposely seek to be surrounded in extreme situations, like entering a war zone, often through an institutionalized and organized approach. In both World Wars, the U.S. government sent artists abroad to the battlefield as an “effort to create a visual record of the American military experience” (U.S. Army Center of Military History, 2020). These are examples of the exceptional resiliency of our species, as well as our drive to create despite the situation. Space, even as an extreme environment, holds no exception for artmaking.

Adopting methods and insights from social sciences, art, and creative practices enables a more robust understanding of topics relating to human behavior in zero gravity. The approach taken to understanding or solving an issue directly affects the insight gained. Sociocultural consideration in current space exploration would make future space activity more inclusive and accessible. Currently most people on the International Space Station tend to be westerners or people from more industrialized societies, it would be fruitful to enable creative and artistic collaboration with individuals of different cultural backgrounds on board the station and on future space missions.

The issue of human space exploration is an intricate challenge that imperatively needs to include the social sciences and humanities. To date, a requirement to apply for an astronaut position at any major space agency is a graduate degree in a STEM field. This excludes philosophers, artists, and architects, who can also provide a more holistic perspective on technical and scientific issues if sent to space. Complex situations that are encountered during space activities require an interdisciplinary response, thus the presence of non-STEM individuals in space should be deemed necessary.

Maintaining a diverse population in not only astronauts, but in anyone involved in space activities is crucial. Currently, women make up 11% of astronauts and 20% of the space workforce (United Nations Office for Outer Space Affairs, 2020). Resulting from a historical prejudice against women engaging in scientific endeavors and space exploration, this imbalance diminishes opportunities for innovation. Diversity and gender equality in space activities is necessary to develop an inclusive space culture with equal access to space and benefits of its exploration.

### Advocacy for Artists in Space Activities

The use and practice of art in space is vital for provoking and enhancing interdisciplinary collaborations, as art in itself is advantageous in scientific inquiry (Simon, 2001). Various Nobel laureates and well-established scientists have been highly involved with art or come from multi-disciplinary backgrounds, which has contributed to their success (Root-Bernstein et al., 2008).

Starting artists residencies either on the International Space Station or in any other context beyond the Kármán boundary is necessary, despite any difficulty in assessing its initial impact. Immersing artists in a novel situation with people of different expertise and backgrounds entails different approaches to interaction compared to designing and sending artworks to space. Top-tier companies and institutes have embraced the idea of their own artists residencies, including the Search for Extraterrestrial Life Intelligence (SETI) institute, SpaceX, Planet Labs, and SolidWorks. NASA and ESA have had a handful of artists in residence. Creative projects from these collaborations have resulted in new scientific discovery; some intentional, like Kitsou Dubois’ weightless choreography that dealt with body awareness in zero gravity and training for astronauts and dancers alike (Bureaud and Dubois, 2005), and some incidental, like Eduardo Kac’s paper airplanes that demonstrated air movement in zero gravity (Kac, 2017; Rose, 2017).

The conditions for creativity and artmaking in space require innovative efforts that require expertise from disciplines outside Science, Technology, Engineering, Arts, and Mathematics (STEAM), such as the social sciences and the humanities. Applying social sciences in extreme environments could contribute to creative processes that are a part of environmental or architectural design, for example the inclusion of proxemics in these designs. Proxemics, also known as personal space, is the “interrelated observations and theories of man’s use of space as a specialized elaboration of culture” (Hall, 1966, p. 1). People from certain cultures prefer to socialize while being physically close to other people, while this may cause people from another culture to feel uncomfortable. According to this theory, the design of an environment may promote or neglect socialization, depending on where the person is from. It is central that designers of vessels or housing in extreme environments consider cultural nuances and social variability to promote better living and working spaces.

### Suggestions for Future Research

Since space art is a relatively new topic, mostly due to the novelty of space exploration itself, we call for the development to further investigate creativity and art in space. The topic can be discussed from a generalistic perspective, regarding the development of a new field, however, in time it may develop into several branches and subfields that may need their own experts. Such was the case for disciplines like space anthropology, a recent field that arose due to the current technological and academic context. In the near future, we expect to see other examples that are specialized with a space focus, including but not limited to the architecture, culinary arts, fashion, and performance art. We are only in the first stages of space art and it will continue to mature and develop. Nevertheless, pioneering work with artists cannot evolve without the collaboration of scientists and engineers. This dependency remains reciprocal, as without artists there is less progress.

As space is a new venture and only over 500 people have traveled outside the Earth’s atmosphere, humankind still has some years before it starts seeing shifts in space society. Conversely, human civilization is accelerating efforts to explore the space tourism industry by establishing a permanent base on the Moon this decade. The addition of sociocultural studies to space ventures may support a humane expansion of society in the years to come.

Aside from expanding the human experience, art has inherent pragmatic characteristics enabling its use as a tool, as is the case of mental health professionals for art therapy. Art therapy is an applied discipline that seeks to enable creative processes as a channel to alleviate stress, anxiety, depression, and other troubling psychological situations for both neurotypical and neurodivergent individuals (Malchiodi, 2012; American Art Therapy Association, 2017). In the case of space art, it is clear that it not only represents a person’s creative potential but may have therapeutic effects that could aid in coping with high-stressful situations in space. Since social isolation is currently unavoidable while in space and gradually decreases performance (National Aeronautics and Space Administration, 2011), art therapy could benefit individuals having trouble cooperating with on-board colleagues or on-ground support staff.

Art practices in space can support strong relationships between astronauts from different cultural backgrounds through collaboration in an unstructured, non-threatening experience (Pietronigro, 2004). Astronauts playfully interacted with Cosmic Dancer (Woods, 1993a), a sculpture designed for zero gravity, taking a break from being scientists and engineers to observe and experience the artwork. The astronauts reported positive shifts in mood, a reevaluation of the environment, and new ideas that were derived from the experience (Woods, 1993b). Making or interacting with art in, not just space but an extreme environment, can have profound effects on the individual’s experience. While we acknowledge that connecting the space art taxonomy that we propose along with our approaches in cognition and creativity might only scratch the surface of this conundrum, we recognize that the domain of creative processes in the extreme environment of space is still in its infancy. Our creative nature as humans helps us redesign our limits and expand our notion of what is possible far beyond Earth’s atmosphere.

## Author Contributions

KH and RM conceived and developed the concept for the manuscript. All authors contributed to writing the manuscript and approve this manuscript for publication and certifies that this material has not been and will not be submitted to or published in any other publication before its appearance.

## Conflict of Interest

The authors declare that the research was conducted in the absence of any commercial or financial relationships that could be construed as a potential conflict of interest.
